# A Mobile App to Improve Symptom Control and Information Exchange Among Specialists and Local Health Workers Treating Tanzanian Cancer Patients: Human-Centered Design Approach

**DOI:** 10.2196/24062

**Published:** 2021-03-23

**Authors:** Robert S Morse, Kaley Lambden, Erin Quinn, Twalib Ngoma, Beatrice Mushi, Yun Xian Ho, Mamsau Ngoma, Habiba Mahuna, Sarah B Sagan, Joshua Mmari, Susan Miesfeldt

**Affiliations:** 1 Da Vinci Usability Lexington, MA United States; 2 Dimagi, Inc Cambridge, MA United States; 3 Muhimbili University of Health and Allied Sciences Dar es Salaam United Republic of Tanzania; 4 Ocean Road Cancer Institute Dar es Salaam United Republic of Tanzania; 5 Maine Medical Center Scarborough, ME United States

**Keywords:** mobile health, mHealth, user-centered design, palliative care, pain, cancer, sub-Saharan Africa, mobile phone

## Abstract

**Background:**

Improving access to end-of-life symptom control interventions among cancer patients is a public health priority in Tanzania, and innovative community-based solutions are needed. Mobile health technology holds promise; however, existing resources are limited, and outpatient access to palliative care specialists is poor. A mobile platform that extends palliative care specialist access via shared care with community-based local health workers (LHWs) and provides remote support for pain and other symptom management can address this care gap.

**Objective:**

The aim of this study is to design and develop mobile-Palliative Care Link (mPCL), a web and mobile app to support outpatient symptom assessment and care coordination and control, with a focus on pain.

**Methods:**

A human-centered iterative design framework was used to develop the mPCL prototype for use by Tanzanian palliative care specialists (physicians and nurses trained in palliative care), poor-prognosis cancer patients and their lay caregivers (patients and caregivers), and LHWs. Central to mPCL is the validated African Palliative Care Outcome Scale (POS), which was adapted for automated, twice-weekly collection of quality of life–focused patient and caregiver responses and timely review, reaction, and tracking by specialists and LHWs. Prototype usability testing sessions were conducted in person with 21 key informants representing target end users. Sessions consisted of direct observations and qualitative and quantitative feedback on app ease of use and recommendations for improvement. Results were applied to optimize the prototype for subsequent real-world testing. Early pilot testing was conducted by deploying the app among 10 patients and caregivers, randomized to mPCL use versus phone-contact POS collection, and then gathering specialist and study team feedback to further optimize the prototype for a broader randomized field study to examine the app’s effectiveness in symptom control among cancer patients.

**Results:**

mPCL functionalities include the ability to create and update a synoptic clinical record, regular real-time symptom assessment, patient or caregiver and care team communication and care coordination, symptom-focused educational resources, and ready access to emergency phone contact with a care team member. Results from the usability and pilot testing demonstrated that all users were able to successfully navigate the app, and feedback suggests that mPCL has clinical utility. User-informed recommendations included further improvement in app navigation, simplification of patient and caregiver components and language, and delineation of user roles.

**Conclusions:**

We designed, built, and tested a usable, functional mobile app prototype that supports outpatient palliative care for Tanzanian patients with cancer. mPCL is expressly designed to facilitate coordinated care via customized interfaces supporting core users—patients or caregivers, LHWs, and members of the palliative care team—and their respective roles. Future work is needed to demonstrate the effectiveness and sustainability of mPCL to remotely support the symptom control needs of Tanzanian cancer patients, particularly in harder-to-reach areas.

## Introduction

Cancer is a growing public health concern in sub-Saharan Africa, with at least 500,000 annual deaths in recent years; a doubling of cancer incidence and mortality is projected by 2030 [1-4]. Although data for Africa as a whole are limited, a study in South Africa and Uganda showed unnecessary distress among late-stage patients with cancer who most often reported uncontrolled pain (87.5%), low energy (77.7%), sadness (75.9%), drowsiness (72.3%), and worry (69.6%), with pain as the most severe symptom [5].

Due to the limited pool of palliative care specialists and low public and private investment in cancer control, there is an urgent need for novel, sustainable, and community-based solutions to address inadequate specialty palliative care services throughout Africa [6-8], with a focus on the four pillars of the World Health Organization (WHO): (1) appropriate policies, (2) education (professional and lay), (3) drug availability, and (4) implementation throughout society [1,9]. These are only achievable with high-quality research to ensure that public health solutions are evidence-based, culturally sensitive, feasible, responsive, effective, and scalable [1].

With the increasing adoption of mobile technology, there is great potential to improve outpatient cancer symptom management through remote access to palliative care. In Tanzania, cell phone ownership increased from 10% in 2002 to 73% in 2015, and smartphone ownership increased from 8% to 13% between 2013 and 2017 [10]. Coupled with a projected further increase in smartphone ownership, mobile health (mHealth, ie, “the use of mobile and wireless devices to support the achievement of health objectives”) [11] promises to grow access to palliative care specialists (hereafter, specialists), resulting in improved symptom management among patients with cancer in Tanzania, our study setting, and other low-resource settings [12]. Although active (ie, mobile survey assessments and digital journaling) or passive (ie, wearables) collection of symptoms over time for a patient with cancer may be a feasible and reliable way of assessing quality of life remotely, there is limited knowledge about the effects of these emerging technologies relative to care coordination, with little effort in low-resource countries [13,14].

A systematic review of existing mobile and web apps focused on pain control in a range of medical conditions, including cancer, reported that although the number of such apps is growing, none of the apps described in scientific databases were available commercially. Furthermore, among the 283 pain control–focused apps identified in the 5 app stores (including Google Play and Apple App Store), scientific evidence of efficacy was nonexistent [15].

A more recent systematic review examined available full-text publications on mobile apps with the following characteristics: focused on cancer pain, downloaded and registered on either a mobile phone or computer, using a numeric scale to assess pain, reporting patient follow-up for more than a week, and available in English. Of the 13 studies reviewed, 5 were randomized controlled trials. The results of this review revealed that app-supported pain control is generally effective in the high-resource setting. Specifically, among the randomized controlled studies reviewed, patients who used the tested apps had less pain than patients without access to the apps. Other outcomes, such as quality of life, pain catastrophizing, and pain self-efficacy, were also improved in app users versus in those from control groups [16]. Existing mobile apps used in cancer pain management often offer a range of functions and specifications, including shared records, training, and real-time feedback. They educate patients about pain and enable documentation of the type of pain experienced as well as feedback regarding symptoms [16]. Importantly, mHealth facilitates pain management among individuals living in rural communities and supports control of other symptoms, including depression [17].

Few studies have examined mobile palliative care solutions in low-resource countries among those with noncommunicable diseases (NCDs), despite increasing awareness of the symptom control needs of patients with chronic diseases, including patients with cancer, and knowledge that by 2030, NCDs will be more prevalent than communicable diseases [12,18-30]. Limitations to previous studies include small sample sizes and varied follow-up times, including some as short as 14 days. There has been a call for larger samples and longer randomized controlled trials as well as further assessment of app functions most critical to symptom control [16]. Notably, previous studies have included limited involvement of health care providers in the design and development of apps, a limitation likely influencing the utility of these technologies [15,19,20]. In close partnership with Tanzanian target end users (physicians and nurses trained in palliative care, patients and lay caregivers [caregivers], and local health workers [LHWs]), we employed a human-centered design (HCD) framework to design, develop, and validate the usability of the mobile-Palliative Care Link (mPCL) prototype; a web and mobile app focused on symptom assessment and control for Tanzanian poor-prognosis patients with cancer that extends access to a limited pool of specialists through partnerships with community-based LHWs.

## Methods

### Overview

This study was approved by the Institutional Review Board of the Muhimbili University of Health and Allied Sciences (MUHAS, Dar es Salaam, Tanzania). Signed informed consent was obtained from all participants before enrollment into the study.

**Figure 1:**
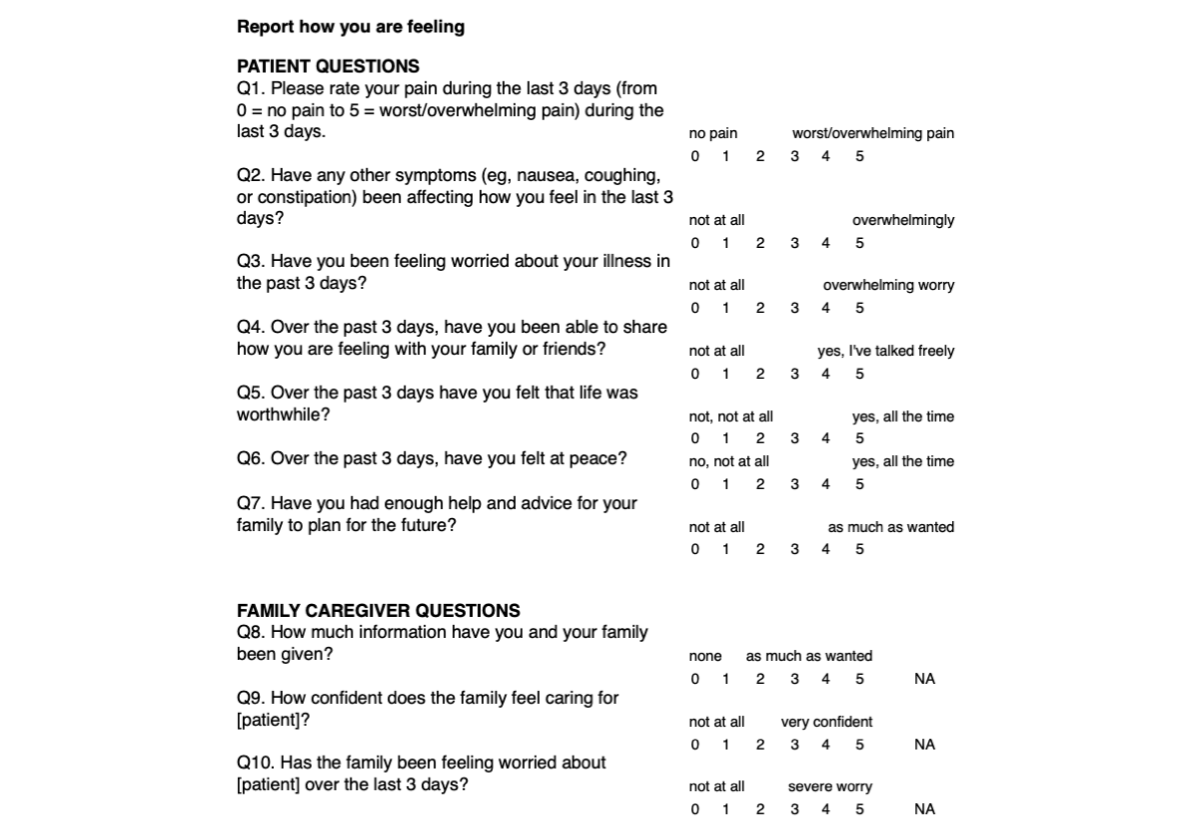
Palliative Care Outcome Scale of the mobile-Palliative Care Link app for a patient and caregiver (for display in Kiswahili). NA indicates that the caregiver is not present.

Using WHO palliative care pillars (policy, education, drug availability, and implementation) as a framework [9] and responses to the validated African Palliative Care Outcome Scale (POS; a 10-item quality of life–focused survey instrument) [31,32] as an outcome measure (Figure 1), we partnered with Dar es Salaam–based Tanzanian specialists (ie, palliative care–trained, Ocean Road Cancer Institute (ORCI)–affiliated oncology physicians and nurses), patients with cancer and caregivers, and LHWs to develop, pilot test, and validate the usability of the mPCL prototype. Located in Dar es Salaam, ORCI is the largest government-supported cancer center in Tanzania.

### Stages of mPCL Development

The app design and development process consisted of six stages.

#### Stage 1: Establishing the Study Team

**Figure 2:**
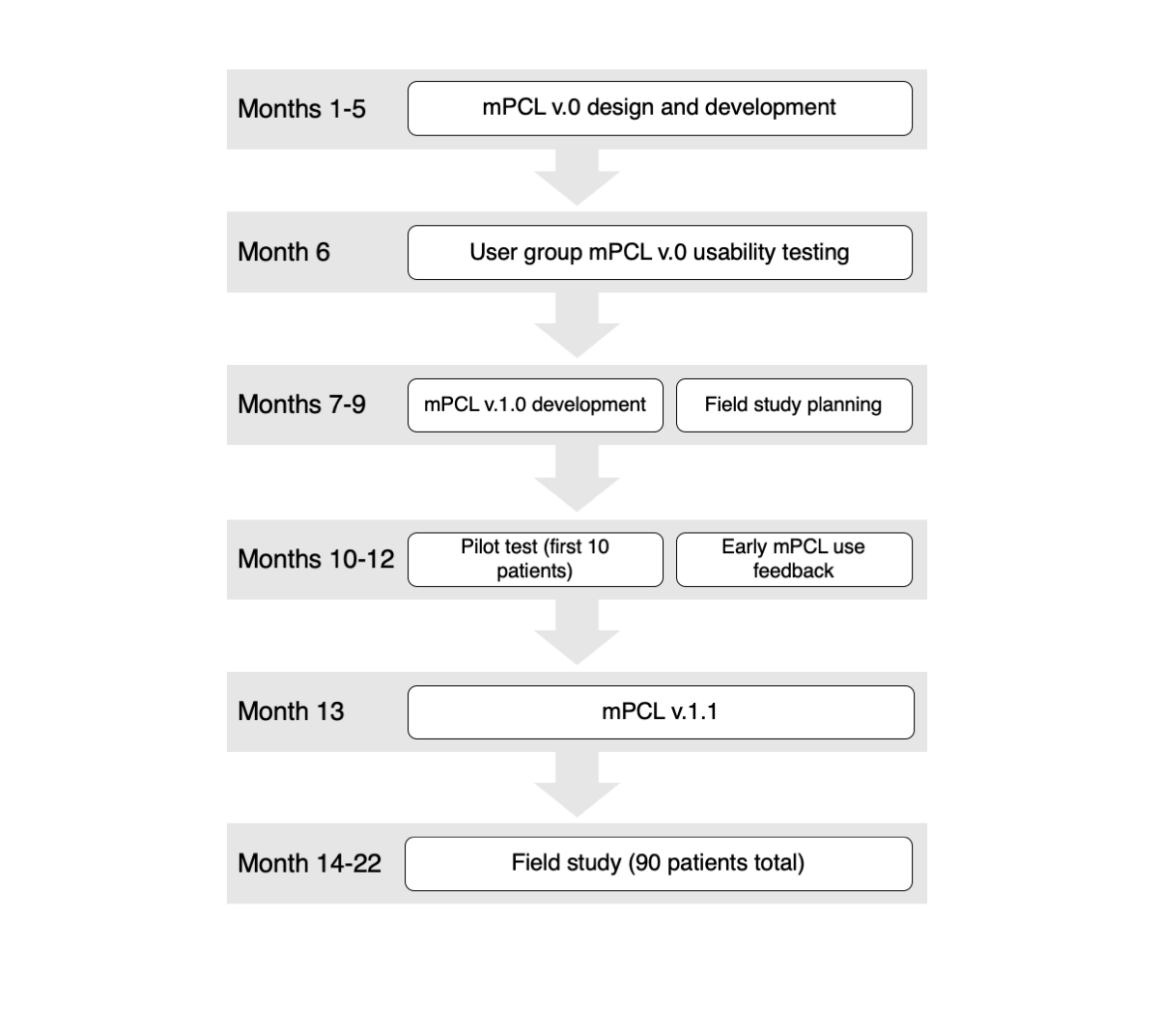
Design and development timeline of the mobile-Palliative Care Link app. Early app use feedback involved only specialists and study personnel; field study results are reported elsewhere.

The multidisciplinary study team established to design and test mPCL included Tanzanian- and US-based partner institutions and organizations—MUHAS, ORCI, Maine Medical Center—and a social software enterprise Dimagi. Team members included Tanzanian and US palliative care specialists, health services researchers, software engineers and designers, and a user experience (UX) specialist (author RM). The team met remotely via videoconference on a monthly basis and communicated via email throughout the app design, development, and testing periods (Figure 2).

#### Stage 2: Defining a Set of App Design Requirements

**Figure 3:**
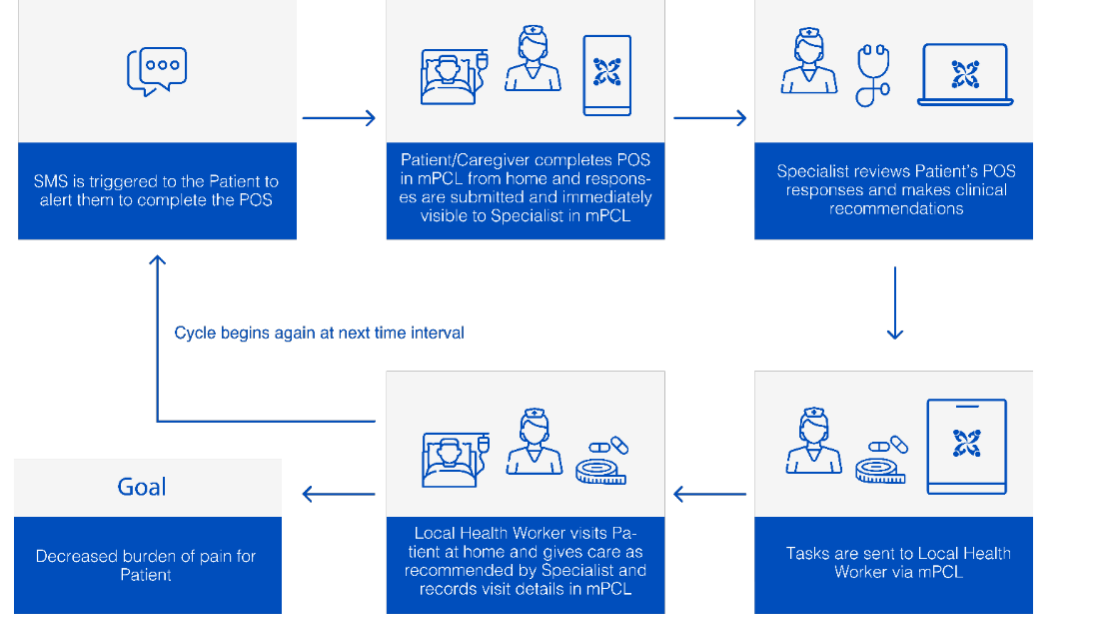
Design of the mPCL app focusing on the patient or caregiver and care team communication and care coordination. mPCL: mobile-Palliative Care Link; POS: Palliative Care Outcome Scale.

The study team defined 3 mPCL design requirements based on a proposed workflow that was endorsed by clinical study team members. Figure 3 shows the care communication and coordination app-facilitated workflow.

Design requirements were as follows:

*Requirement 1: Streamlined and timely collection of patient self-reported symptoms.* Central to mPCL’s utility is its ability to regularly and in real time assess the patient’s quality of life (with a focus on pain control) through scheduled, twice-weekly POS delivery and response collection, including 7 patient-focused items and 3 caregiver-completed items (Figure 1). Permission was secured to adapt this survey instrument for mobile use.

*Requirement 2: Interdisciplinary care coordination. *Following a patient-centric, interdisciplinary system of care coordination, mPCL was designed for access and use by the patient or caregiver and key members of the patient’s clinical care team to deliver responsive, high-quality community-based palliative care services. We define these roles as follows:

1. Specialists include hospital-based specialist physicians (oncologists trained in palliative care) and palliative care nurses (hereafter referred to as nurses). The specialist physician’s primary tasks are to generate a shared synoptic clinical record and palliative care plan, review POS results, and oversee the patient’s care. Nurses support the development of the synoptic clinical record, conduct visits with the patient or caregiver in coordination with the LHW, and serve as a liaison between all care team members as well as an emergency contact for patients or caregivers. As such, both specialist physicians and nurses play a role in creating synoptic clinical records in mPCL. The clinical record and care plan can be viewed by other members of the care team and can be updated at any time by the specialist based on POS responses and input from the nurse, LHW, and patient or caregiver. Specialist physicians have the exclusive ability to prescribe, supply, and make adjustments to morphine and other medications critical to pain and other symptom management.
2. The community-based LHW, located in close proximity to the patient’s or caregiver’s home, assists with regular remote assessment, monitoring, and management of the patient’s symptoms via in-person visits recorded in the form of mPCL follow-up interactions and through mPCL communication or care coordination in partnership with other team members, thereby providing frontline care based on direct ongoing specialist guidance and the needs of the patient.
3. The cancer patient receiving outpatient, home-based palliative care as well as their caregiver, responsible for providing in-home patient support, can access and use the app to complete the 10-item POS and submit results to mPCL’s cloud-based server for subsequent review by the LHW and review or action by a specialist. Additional patient-centric design specifications include a set of low-literacy educational resources developed in Kiswahili (Tanzania’s primary language) with a focus on the causes and management of common late-stage cancer symptoms.

*Requirement 3: Symptom response–focused communication between the care team and patient or caregiver*. Shared access to POS results, an evolving synoptic clinical record and palliative care plan, and follow-up notes by care team members on interactions with the patient or caregiver allow for: (1) timely communication to support patient-centered care plan decision making in response to an LHW’s in-person assessment of the patient experiencing escalating symptoms or other needs, (2) coordination of care to implement changes to the care plan, and (3) important updates regarding changes in patient status (ie, hospitalization or death). mPCL also provides patients with ready access emergency phone contact with a specialist or LHW in the event of rapidly escalating symptoms or acute changes in clinical status.

#### Stage 3: Defining User Requirements

In accordance with HCD methodology, user characteristics and needs were gathered and analyzed to create a set of user requirements before app development [33]. The first step in doing this was to create a set of user personas. Each persona presented a summary of the key characteristics, background, and needs of a representative user from each user group. The personas were developed by a subset of the study team and reviewed by all team members, including those in Tanzania who had the closest understanding of prospective app users. After the personas were complete, a set of user stories was created. Each user story described how one or more users (represented by the personas) would use the app, either on their own or together. The user stories functioned as a means to clearly and concisely define how the app would be used by each user group. As with the personas, the user stories were reviewed by all team members.

#### Stage 4: Creating the App Prototype, mPCL v.0

The app prototype, mPCL v.0, was designed and built to directly support the core requirements defined in Stage 2, summarized above. The process of designing and creating the prototype involved (1) drafting the data architecture, (2) creating display pages and input forms to populate with content, and (3) defining custom user interfaces and permissions for types of users based on their specific roles and tasks. Periodic technical reviews and audits were conducted internally by Dimagi to ensure app design optimization and to verify technical requirements for data collection and analysis, for example, configuring unique user names and updating a change in medication. The back and front ends of the functional prototype were built and prepared by Dimagi staff with feedback from clinical team members and the team’s UX specialist. mPCL was developed on CommCare; Dimagi’s open-source, secure, cloud-based case management platform that allows end users to collect data and deliver interventions via custom-built mobile and web apps. This enabled the team to rapidly iterate the design and prepare a functional prototype for testing and use [34].

#### Stage 5: Expert UX Review

Once the prototype was complete, an expert review was conducted by the UX specialist. The goal of this review was to identify and fix areas where the app was not in compliance with established UX best practices. The UX specialist ascertained areas of improvement and suggested changes. These changes were reviewed by team members and implemented into an updated version of the app. Key improvements included reorganizing the workflow in the app to make it easier for specialists and LHWs to access patient information, changes to the set of data shown for each user group to ensure availability of all essential information, and updates to labels and terminology to improve clarity and ease of use.

#### Stage 6: Testing the Prototype

Usability and pilot testing were conducted with target end users, whereby the mPCL prototype was iterated and further developed after each phase of testing.

##### Usability Test

mPCL v.0 usability was assessed in individualized in-person usability testing sessions conducted by the UX specialist with participants representing target end users. Two site visits in Tanzania were led by US study team members, in collaboration with MUHAS and ORCI partners, to conduct in-person prototype usability testing and then train end users on the system just before the pilot test. The goals of usability testing were to (1) validate the design of mPCL and identify any remaining design issues, (2) uncover opportunities for system improvement, and (3) learn about the target user’s behaviors and prototype app interactions [35]. mPCL v.0 usability testing was conducted with a diverse sample of patients, LHWs, and specialists. Potential participants were identified and recruited by ORCI-based study team members. The eligibility criteria for patients included adult ORCI inpatients with known untreatable cancer. Specialists included ORCI-based oncologists and a palliative care nurse. LHWs were eligible to participate if they were within 50 km of ORCI and had experience caring for ORCI patients. Written informed consent was obtained from all study candidates before the usability testing session.

Usability testing sessions occurred in person in a private space at ORCI. Testing for patients and LHWs was conducted in Kiswahili, with a translator. Testing for specialists was conducted in English. Patients were first briefly trained by a study team member on the basic use of mPCL. They were then asked to use an mPCL-equipped study smartphone to perform the following tasks to assess usability: (1) access educational resources, (2) complete and submit POS responses, and (3) contact a care team member. Specialist physicians were asked to use an mPCL-equipped tablet to (1) set up a mock patient’s synoptic clinical record, including a discharge palliative care plan; (2) review POS results; (3) enter follow-up patient notes, including changes in care plan; and (4) exchange notes with an LHW. These notes were intended to document requests for in-person assessment, collect additional clinical information, convey treatment recommendations and care plan changes. The nurse was asked to (1) register a mock patient into mPCL and enter relevant sociodemographic and clinical information into the synoptic clinical record, (2) complete the POS, (3) record a note documenting an interaction with the patient, (4) review the patient’s POS results and medications, and (5) update the individual’s contact information. LHWs were asked to use a study mPCL-equipped smartphone to (1) review a mock patient record, including POS results and (2) exchange notes with a mock specialist and patient regarding the patient’s assessment and symptom control. All usability testing participants were observed performing the predefined tasks and all issues that arose in performing the tasks (eg, missteps in navigating through the app to perform a task and errors entering data) were documented. Recorded usability issues and participants’ recommendations for changes to the prototype design were reviewed and considered by the study team. At the end of the usability testing, all participants completed a verbally administered survey that assessed users’ perceived mPCL ease or difficulty of use. A set of recommendations was derived from usability testing feedback to inform the iteration of a more robust and user-validated mPCL prototype. App modifications ultimately accepted and employed were based on feasible design decisions with the goal of maximizing usability. The final mPCL v.1.0 prototype was then used in the subsequent pilot test.

##### Pilot Test

As part of a larger prospective field study of the system, cancer patients were enrolled and consented upon planned discharge to home from ORCI and randomized to either the mPCL intervention or twice-weekly phone collection of POS responses by an ORCI-based clinician team member. Here, we describe the pilot testing used to inform the final version of mPCL (v.1.1), deployed and tested in the field study. A full description of the field study and its outcomes will be reported elsewhere (manuscript in preparation). In brief, patient eligibility for both the pilot test and field study included (1) an adult ORCI inpatient with known untreatable cancer, being discharged to home, (2) a 4-month life expectancy or greater per specialist physician assessment, (3) residence within 50 km of ORCI for medication access, (4) caregiver available to support outpatient care for the illness duration, (5) an LHW consented to support the patient’s outpatient care for the test’s duration (up to 4 months post discharge), and (6) completed primary school education. Intervention patients lacking personal smartphones were loaned an Android device with the mPCL app preinstalled and available for use during the 4-month study period, and those with reliable access to their own personal smartphone were provided the option to install and use the mPCL app on their own device. An ORCI information technology specialist working directly with the study team assisted patients with the preparation and maintenance of devices for study purposes (ie, acquisition of SIM cards and installation of mPCL) and served as the first point of contact to respond to any emergent technical issues over the course of the study. For specialists, mPCL was accessible from their office-based computers as a web app as well as their smartphones as a web or native mobile app. LHWs had the option to either use their own mPCL-enabled smartphones or an Android device provided for use during the study with the mPCL mobile app preinstalled.

Real-time feedback and input regarding mPCL use as well as study process or procedure problems were requested via email and during regular team meetings from specialists (physicians and nurse), and ORCI-based study personnel during the 2-month pilot test period. Within this time, a total of 10 patients were enrolled and randomized to mPCL versus phone contact. At the end of the 2-month pilot test period, patient recruitment was held for close to a month during which mPCL use feedback and pilot test process recommendations were compiled, analyzed and used to iterate on and finalize the app prototype, mPCL v 1.1, for further field study.

## Results

### mPCL v.0 Usability Testing

A total of 21 potential target end users participated in mPCL v.0 usability testing: 7 patients and caregivers, 8 specialists, and 6 LHWs. Of the 7 patients who participated, 6 were women (1 man), and none of them reported any secondary school education or spoke English. Patients’ ages ranged from 34 to 64 years, and 2 patients or their caregivers owned an Android smartphone, 4 owned a mobile phone, and 1 did not own a phone. Among specialists, 7 physicians and 1 nurse participated, and 6 of them were women (2 men). Specialists had 4-14 years of clinical experience, were English speaking, and owned Android smartphones. The LHW participants included 4 women and 2 men with 4-14 years of clinical experience; all owned Android smartphones. All LHWs were fluent in Kiswahili, with a few also conversant in tribal languages, and reported limited English language proficiency.

Open-ended user feedback and usability issues identified during usability tests were itemized and reviewed by the mPCL study team for optimization of mPCL v.0 to mPCL v.1.0. This included review of direct feedback from users on role-specific tasks and navigation of custom interfaces, designed for each user role. Design component improvement recommendations were subsequently reviewed, validated and acted upon. Examples of user task performance improvement recommendations prompting app optimization included (1) reformatting the POS assessment for patients and caregivers to display individual items one at a time instead of all on one page, making it easier for those not accustomed to navigating a touchscreen; (2) adding more comprehensive clinical data for collection by specialists, specifically providing more detail on the patient’s current and historical medications; (3) adding more information on the patient’s social history to the clinical record (ie, family support resources, such as their living situation and nutritional support); (4) translating all information displayed to LHWs into Kiswahili to make the app easier to use for those with limited English language proficiency; and (5) adding the individual patient’s cancer type and stage to the patient list display for easier identification. Other emergent themes in user feedback that were acted upon included the need for clearer delineation of user roles and tasks and related user permissions (eg, creation of follow-up notes by a specialist physician and patient enrollment and registration by a nurse) and clarification of POS data collection and monitoring (eg, reminder schedule and modality to prompt patients to submit POS assessments, and notification mechanisms to alert the care team of a new POS submission). As summarized in Table 1, the perception of mPCL’s usability for tasks performed ranged from a low degree of ease and acceptability (3 out of 4) to a very high degree of ease and acceptability (1 out of 4). In general, respondents found mPCL easy to use, with an average usability score of 2 and below for any given task. Of particular note, all LHWs (6 out of 6) reported a high degree of ease and acceptability for time spent reviewing the clinical record and the POS assessments. Several respondents remarked that they anticipated that ease of use and acceptability would improve with increased experience using the app.

**Table 1:** Prototype usability test survey results among patients, specialists, and local health workers (n=21).

Survey item by user group		Response^a^			Number of responses to survey items, *n*(%)
		Mean (SD)	Range		
**Patients (*n*=7)**					
	Ease or difficulty of POS^b^ completion	1.9 (0.9)	1-3	7 (100)	
	Acceptability of time to complete POS	1.3 (0.76)	1-3	7 (100)	
	Ease or difficulty of using educational materials	1.6 (0.79)	1-3	7 (100)	
	Ease or difficulty of making emergency phone calls	1.2 (0.41)	1-2	6 (86)	
**Specialists (*n*=8)**					
	Ease or difficulty of creating a clinical record	1.5 (0.53)	1-2	8 (100)	
	Acceptability of time spent creating a clinical record	1.3 (0.49)	1-2	8 (100)	
	Ease or difficulty of reviewing a clinical record	1.1 (0.35)	1-2	8 (100)	
	Acceptability of time spend reviewing a clinical record	1.3 (0.49)	1-2	7 (88)	
	Ease or difficulty of reviewing POS	1.6 (0.55)	1-2	5 (63)	
**Local health workers** **(*n*=6)**					
	Ease or difficulty of reviewing a clinical record	1.3 (0.82)	1-3	6 (100)	
	Acceptability of time spent reviewing a clinical record	1 (0.0)	1-1	6 (100)	
	Ease or difficulty of reviewing POS	2 (0.63)	1-3	6 (100)	
	Acceptability of time spent reviewing POS	1 (0.0)	1-1	6 (100)	
	Ease or difficulty of recording a patient interaction	1.8 (0.5)	1-2	4 (67)	

^a^All survey item responses were scored from 1 to 4, with 1=very high degree of ease or acceptability and 4=very low degree of ease or acceptability.

^b^POS: Palliative Care Outcome Scale.

### mPCL v.1.0 Pilot Test

During the 2-month mPCL pilot test period, 4 specialist physicians, 1 nurse, 5 LHWs, and 10 patients who were randomized to mPCL use versus phone-contact POS collection were enrolled. Specialists, including a subset of mPCL users who were also study team members (coauthors TN, BM, HM, and MN) were asked to provide real-time feedback on issues or questions related to either mPCL v.1.0 use or study processes and procedures, in preparation for the subsequent field study. Table 2 summarizes examples of issues identified; some of this feedback required immediate resolution, whereas other feedback was addressed at the end of the pilot test period.

**Table 2:** Examples of mPCL pilot test feedback and corresponding actions taken.

Issue identified	Action taken
CommCare failed to recognize installation codes necessary to install the mPCL^a^ app on a study phone	ORCI^b^-based team instructed on resolution
CommCare failed to install updates	ORCI-based team instructed on resolution
LHWs^c^ and patients unknowingly uninstalled mPCL or reset cellular internet settings	ORCI-based information technology support team member engaged to address issues on demand
Study nurse could not complete the mPCL clinical record if the cancer stage was not known	“Unknown” was added as a response selection
Patients requested to use their own personal SIM cards rather than using SIM card provided by study	Personal SIM cards were allowed and used with study phones, with participant’s permission
ORCI study team noted variability in the ease of training patients on the use of mPCL	Procedure established to capture data on patient’s mPCL training ease or difficulty (eg, number of times patient training repeated and specific challenges encountered during training)
Difficulty for the patient to select which care team member they wished to contact by phone in emergency setting (ie, nurse, LHW, or specialist)	mPCL adjusted to allow the patient to more easily select the desired care team member
Patients completed more than one Palliative Care Outcome Scale in a given day	Feedback provided to patient that they were submitting duplicate surveys, including a reminder that they could contact a care team member by phone in the event of escalating symptoms

^a^mPCL: mobile-Palliative Care Link.

^b^ORCI: Ocean Road Cancer Institute.

^c^LHWs: local health workers.

### mPCL v1.1 Prototype Finalization

**Figure 4:**
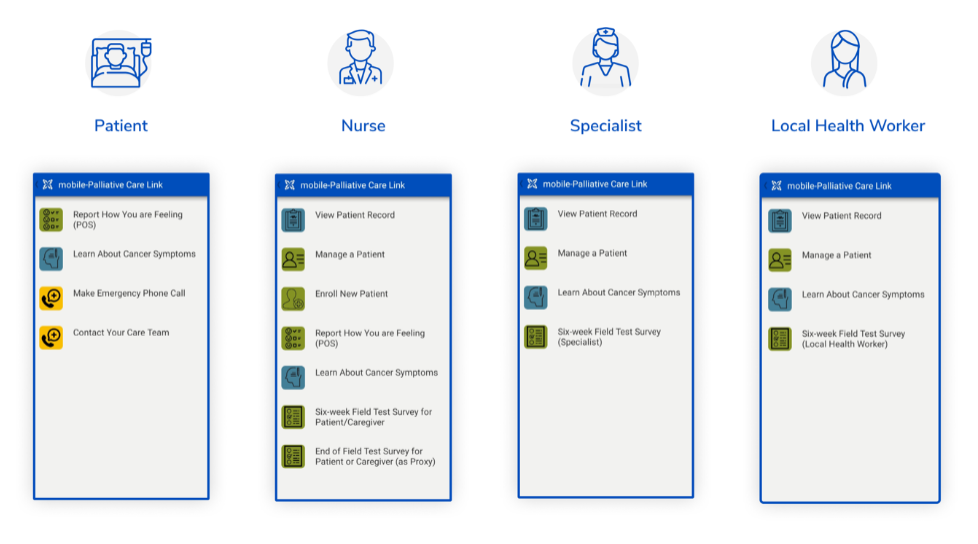
Four separate interfaces for four different user roles (from left to right): patient or caregiver, nurse, specialist physician, and local health worker.

The mPCL v1.1 prototype functionalities summarized below were focused on real-time symptom assessment and care coordination, with the primary aim of effective symptom management and maintenance of quality of life. For the purpose of the mPCL field study, we defined and validated 4 individual mPCL user groups with interfaces, access, and permissions to functionalities specific to each group’s roles and tasks in managing study patients: specialist physician, nurse, LHW, and patient or caregiver. Specifically, a nurse was determined to be the only user group with the ability to register a new study patient in mPCL via the Enroll New Patient module. Additional tasks assigned to the nurse to support field study-specific activities (eg, ability to drop a patient from the study) were built and validated via the pilot test, in preparation for the subsequent field study. Field study-specific surveys for each user group were directly included and disseminated to users through the app (Figure 4 shows the screenshots of user interfaces).

#### POS

**Figure 5:**
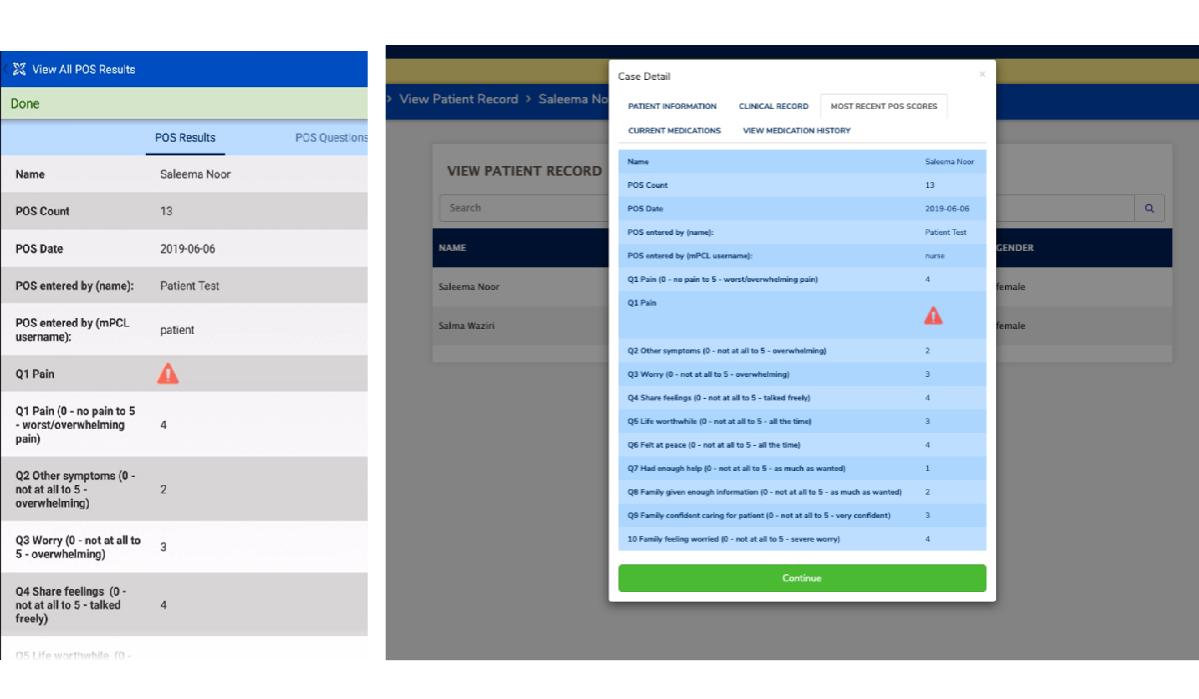
Palliative care Outcome Scale responses (mock patient) as viewed in the mobile app on a smartphone (left) and on the web app (right) by the specialist. A red triangle icon is displayed to alert the care team to reported pain scores that are above the set threshold.

The core priority of mPCL is adequate pain control based on patients’ self-reported POS scores. The 10 Likert-scaled POS items are included in the app as a survey form, with single-choice response options ranked from 0 to 5, corresponding to symptom severity. The assessment is displayed in Kiswahili and designed to collect responses directly from patients and their caregivers. Scores are automatically available for review by the clinical care team upon synchronization of data on mPCL-enabled devices connected to the CommCare cloud server back-end. Through mPCL, patients are reminded via SMS text message to complete the POS on a twice-weekly basis, with results immediately accessible to the specialist, nurse, and LHW for timely tracking and response, as needed. Flags signifying escalating symptom scores were built into the app for more immediate attention from the care team (Figure 5).

#### Synoptic Clinical Record and Palliative Care Plan

**Figure 6:**
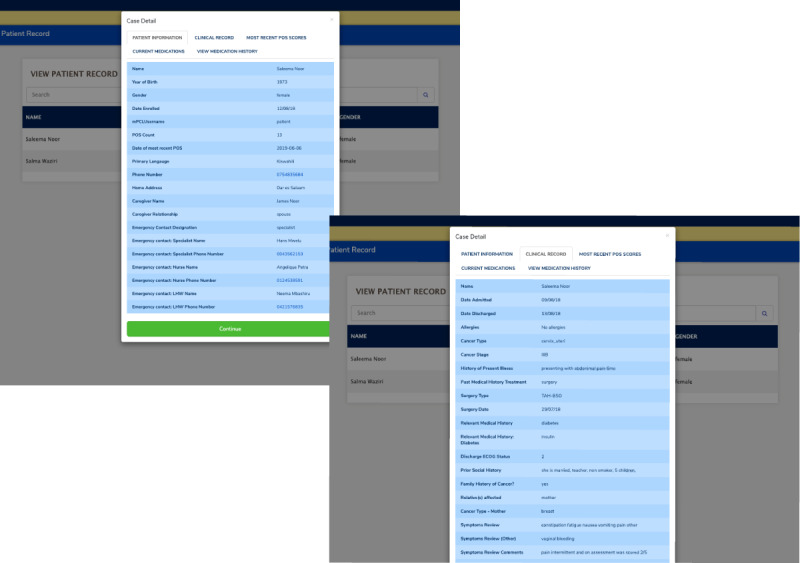
Clinician web app view of patient information (top) and clinical record of the mock patient record (bottom).

A series of templated forms allow specialists to create and share access to a synoptic clinical record and discharge palliative care plan that includes the patient’s basic demographic information, social history, disease type and stage, noncancer comorbidities, a summary of previous cancer treatments, essential imaging and laboratory results, and an outpatient palliative care plan, including discharge medications and allergies. The synoptic clinical record facilitates clinical and social history data collection by a specialist physician and nurse immediately following inpatient hospital discharge of the patient to home for ongoing palliative care coordination. This record is available to both the specialist physician and nurse with read or write access and LHW with read access (Figure 6 shows the examples of parts of the clinical record that are viewable to care team users).

#### Follow-Up Patient Interaction

Postdischarge changes in clinical status, including any communication with or in-home assessment of the patient, readmissions, clinic visits, medication adjustments, or death are recorded in mPCL by a member of the care team using clinical follow-up form templates. This clinical documentation is intended for communication and care coordination among care team members and to update the patient’s clinical record and alert other care team members of important changes in clinical status.

#### SMS Text Messaging and Reminders

One-way SMS text messaging is enabled and programmable through the app to support, for example, scheduled reminders to complete the POS or other study survey instruments.

#### Educational Module

**Figure 7:**
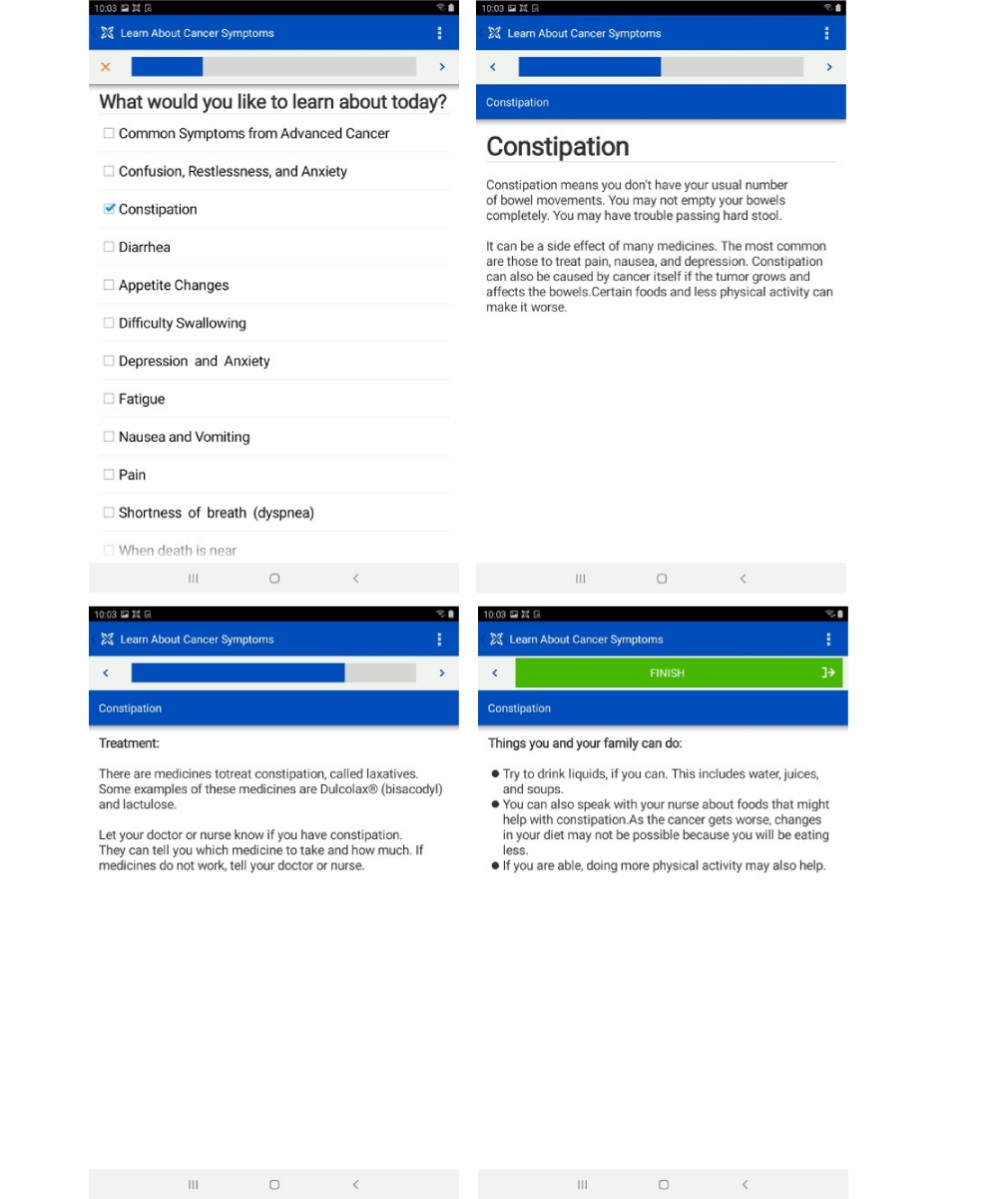
Example screenshots of the patient symptom-focused educational resource (displayed in Kiswahili for patients). Patients are able to select specific content areas they would like to learn more about.

Basic educational information, adapted from publicly available, web-based resources [36,37], was developed to improve the patients’ and caregivers’ awareness of the causes and management of a wide range of late-stage cancer symptoms (ie, pain, nausea, constipation, and shortness of breath). Through the support of a US-based literacy expert, the educational module was developed at a primary school reading level and then translated into Kiswahili with the assistance of MUHAS or ORCI study team members and input from patient or caregiver usability test participants (Figure 7).

#### Emergency Contact

**Figure 8:**
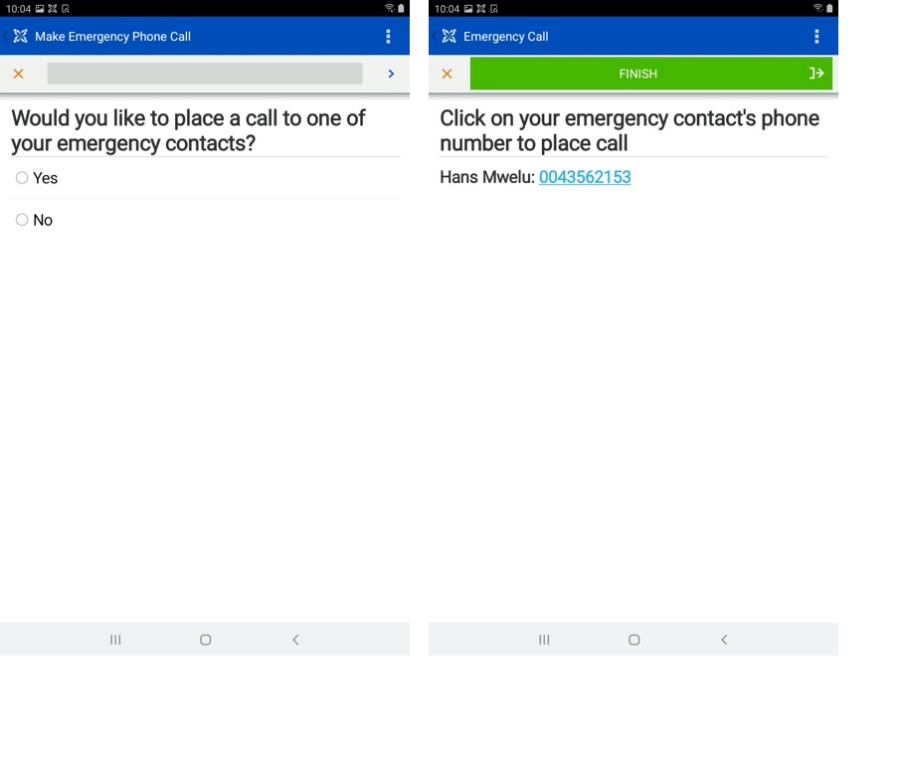
Screenshots of the emergency contact module. When the patient clicks Yes on the first screen of the module (left), they are advanced to the next screen (right), where they can click on a hyperlink to directly call the designated emergency contact.

An emergency contact module was built to enable patients or caregivers to directly connect with a member of the care team via phone (Figure 8).

## Discussion

### Principal Findings

Here, we describe mPCL HCD and development processes. This secure, patient-centered web and mobile app is focused on extending the reach of a limited pool of specialist clinicians. Specifically, mPCL facilitates real-time symptom collection and reporting for direct communication between patients or caregivers and their clinical care team members, and LHW-specialist care coordination to support prompt and effective community-based symptom control. Through the work described here we show that mPCL is usable and feasible for executing and fulfilling tasks specific to and expected of each user role.

Although this is not the first mobile app dedicated to cancer-related pain and other symptom control, to our knowledge; this is the first such system developed expressly to support palliative care in low-resource settings using a community-based framework of care. Critical to the mPCL design process was input collected directly from potential target end users to inform prototype iterations before finalization of a version deployed in a real-world clinical setting. Although smartphone ownership and connectivity have greatly increased across Tanzania, more of this growth is among younger, more educated, and affluent populations [10], and the use of a smartphone app to deliver a symptom control intervention at a population level in Tanzania has only recently emerged as an area of exploration for researchers and developers. As such, a full awareness and understanding of the target population’s context is first needed to build a usable app in terms of access to technologies and resources, ability to adopt and effectively use the intervention, and preferences regarding design, to include a careful assessment of the cultural competency of the app in different populations and settings, especially those facing the greatest socioeconomic and geographic barriers to care.

As with mPCL, individual apps can offer a wide range of functions and specifications, such as educational resources, diaries, reminders, treatment recommendations, and real-time communication with health care providers. There is a need for comprehensive reporting and testing of these individual functions and features to examine which components are most helpful in symptom control. Furthermore, standardized quantification of patient-reported symptoms can be lacking or limited in apps. There has been a call for standardized protocols and tools for pain (and other symptom) assessment, as this would strengthen future mHealth studies and allow investigators to synthesize and compare results of individual studies in a range of settings and populations [18]. Here, we designed and demonstrated the usability of an app directly addressing pain and other symptoms (physical and emotional) using a validated patient- and caregiver-focused tool (ie, the African POS). We assessed the POS and other mPCL functions, as well as user-focused features among representatives of all target user groups.

Cancer-related pain is a global issue, with the greatest concern among those in underresourced settings where access to specialists and other resources, including medications, is limited or nonexistent. mPCL is focused on pain and other acute and chronic late-stage symptoms, directly linked to quality of life, among patients with cancer from a low-resource sub-Saharan African country. The design of an interface and functionalities specific to the role of and usability tested among each member of the palliative care team promises to address some of the limitations cited in previous work in this area. Critical to the utility and usability of mPCL was attention to the unique cultural, sociodemographic, and educational experiences (including language proficiencies) and backgrounds of patients and their caregivers. We adopted a rigorous HCD approach with active engagement and participation of patients and caregivers throughout the app design process—a technique that is viewed as essential to the adoption and ultimate effectiveness (herein, reflected in improved quality of life among patients with cancer) of new technologies in low-resource settings [38-40].

Core to mPCL functionality is the scheduled delivery and collection of patient symptoms and quality of life indicators made available real time to all care team members. This functionality promises to deliver a prompt response to escalating symptoms. The clinician end users viewed immediate access to the synoptic clinical record and follow-up notes, as well as functions focused on user group communication and care coordination, as critical. The generation and tracking of the synoptic clinical record were not found to be cumbersome among the specialists who participated in both initial usability testing and early pilot testing and users perceived the clinical information to be up to date. In line with previous literature revealing that real-time communication improves outcome relative to adequate pain control, access to emergency phone contact with a care team member was seen as an essential component of mPCL [16].

Notably, both patients and LHWs reported the importance of the educational module in improving awareness of anticipated late-stage, cancer-associated symptoms as well as an understanding of the basic means to control these symptoms. Although these resources were directed at the patient and caregiver, LHWs reported that this information was informative for them personally and they believed that it supported them in their care of patients with cancer.

### Conclusions

Here, we describe the design and development of a mobile app aimed at extending the reach of a limited pool of specialists, dedicated to symptom control and improved quality of life among late-stage cancer patients in low-resource settings. We followed an HCD framework with direct engagement of all target end user groups—specialists (physicians and nurses), LHWs, and patients and caregivers—to design the prototype. The central focus of the app was real-time symptom monitoring (using a validated scale) and communication. Usability testing revealed general app acceptance, and early pilot testing showed the app to be usable and feasible in the setting of a single urban cancer institute. Our broader, randomized field study will provide further evidence regarding the clinical utility of mPCL. Additional questions related to this work include the generalizability of mPCL to other geographic settings and in settings with less access to symptom control medications and other support resources. As mobile technologies continue to grow and evolve in low-resource settings such as Tanzania, the field of cancer medicine can greatly benefit from an understanding of how to build patient-centric tools optimized for remote symptom monitoring and tracking as well as effective and efficient care coordination. Furthermore, rigorous studies of the use of tools such as mPCL in practice will be critical to understanding how they can be widely adopted and scaled.

## Abbreviations

HCD: human-centered design
LHW: local health worker
mHealth: mobile health
mPCL: mobile-Palliative Care Link
MUHAS: Muhimbili University of Health and Allied Sciences
NCD: noncommunicable disease
ORCI: Ocean Road Cancer Institute
POS: Palliative Care Outcome Scale
UX: user experience
WHO: World Health Organization
